# Supplementary material for: Exploring the Cost-Effectiveness of Newborn Screening for Metachromatic Leukodystrophy (MLD) in the UK
Source: Int J Neonatal Screen. 2024 Jun 26;10(3):45. doi: 10.3390/ijns10030045 (PMC11270184; doi:10.3390/ijns10030045)
Supplement: Supplementary file 1 [file IJNS-10-00045-s001.zip › IJNS-2931165-supplementary.pdf]

Supplementary information

For patients receiving best supportive care - the following transition probability formulae have been used to estimate transition probabilities with a constant hazard between each of the GMFC-MLD stages 1–6. The transition rate is defined as '1-(1/mean time in state)' and can be converted into the transition probability for the model cycle length using the following formula:

$$Tp_{cycle\ length} = 1 - \exp\left(-(transition\ rate) * cycle\ length\right)$$

Thus, the following formula was applied to calculate the transition probability assuming a constant hazard when using the mean time in state input value:

$$Tp_{cycle\ length} = 1 - \exp\left(-\left(\frac{1}{mean\ time\ in\ state}\right) * cycle\ length\right)$$

An example for the transition from GMFC 0 to 1 is provided below:

Monthly transition probability from GMFC-MLD 0 to GMFC-MLD 1 = 1- (1- 0.5)^(1/[Average or median Rate of Progression from GMFC-MLD 0 to GMFC-MLD 1])

Table S1: Late Infantile MLD – BSC transition probabilities:

	GMFC-MLD 0	GMFC-MLD 1	GMFC-MLD 2	GMFC-MLD 3	GMFC-MLD 4	GMFC-MLD 5	GMFC-MLD 6	Dead
GMFC-MLD 0	0.73858	0.26142	0	0	0	0	0	0
GMFC-MLD 1	0	0.76490	0.23510	0	0	0	0	0
GMFC-MLD 2	0	0	0.76427	0.23573	0	0	0	0
GMFC-MLD 3	0	0	0	0.76427	0.23573	0	0	0
GMFC-MLD 4	0	0	0	0	0.764274	0.23573	0	0
GMFC-MLD 5	0	0	0	0	0	0.901197884	0.09880	0
GMFC-MLD 6	0	0	0	0	0	0		
Dead	0	0	0	0	0	0	0	1

Table S2: Early Juvenile MLD – BSC transition probabilities:

Note the transition probabilities for the pre symptomatic early juvenile and early symptomatic BSC engines are the same, it is the age at entry into the model and the presence of symptoms at baseline that differ).

	GMFC-MLD 0	GMFC-MLD 1	GMFC-MLD 2	GMFC-MLD 3	GMFC-MLD 4	GMFC-MLD 5	GMFC-MLD 6	Dead
GMFC-MLD 0	0.89908	0.10092	0	0	0	0	0	0
GMFC-MLD 1	0	0.94680	0.05320	0	0	0	0	0
GMFC-MLD 2	0	0	0.76427	0.23573	0	0	0	0
GMFC-MLD 3	0	0	0	0.76427	0.23573	0	0	0
GMFC-MLD 4	0	0	0	0	0.764274472	0.23573	0	0
GMFC-MLD 5	0	0	0	0	0	0.964563099	0.03544	0
GMFC-MLD 6	0	0	0	0	0	0		
Dead	0	0	0	0	0	0	0	1

Clinical trial data demonstrate that for those arsa-cel treated patients that experience disease progression (unstable partial responders only), the rate at chich they progress is considerably slower than the natural history. The transition probabilities are therefore calculated as follows:

$$Tp_{cycle\ length} = 1 - \exp\left(-\left(\frac{1}{(mean\ time\ in\ state\ for\ BSC * arsa - cel\ rate\ of\ progression)}\right) * cycle\ length\right)$$

An example for the transition from GMFC 2 to 3 is provided below:

*Monthly transition probability from GMFC-MLD 2 to GMFC-MLD 3 = 1- (1- 0.5)^(1/[Average or median Rate of Progression from GMFC-MLD 2 to GMFC-MLD 3 in BSC arm * arsa-cel rate of progression from GMFC-MLD 2 to 3])*

Table S3: Late Infantile MLD – arsa-cel unstable partial responder transition probabilities

Transitions from GMFC-MLD 0 to 1 are equivalent to BSC as a progression modifier for this transition is not calculable.

**Unstable partial responders**	GMFC-MLD 0	GMFC-MLD 1	GMFC-MLD 2	GMFC-MLD 3	GMFC-MLD 4	GMFC-MLD 5	GMFC-MLD 6	Dead
GMFC-MLD 0	0.73858	0.26142	0	0	0	0	0	0

GMFC-MLD 1	0	0.76490	0.23510	0	0	0	0	0
GMFC-MLD 2	0	0	0.76427	0.23573	0	0	0	0
GMFC-MLD 3	0	0	0	0.76427	0.23573	0	0	0
GMFC-MLD 4	0	0	0	0	0.764274	0.23573	0	0
GMFC-MLD 5	0	0	0	0	0	0.901197884	0.09880	0
GMFC-MLD 6	0	0	0	0	0	0		
Dead	0	0	0	0	0	0	0	1

Table S4: Early juvenile MLD – arsa-cel unstable partial responder transition probabilities

Any PS-EJ and ES-EJ treated patient that experienced disease progression despite treatment contributes to the calculation of the transition probabilities for the unstable partial responder group. Transitions from GMFC-MLD 0 to 1 are equivalent to BSC as a progression modifier for this transition is not calculable.

**Unstable partial responders**	GMFC-MLD 0	GMFC-MLD 1	GMFC-MLD 2	GMFC-MLD 3	GMFC-MLD 4	GMFC-MLD 5	GMFC-MLD 6	Dead
GMFC-MLD 0	0.89908	0.10092	0	0	0	0	0	0
GMFC-MLD 1	0	0.95476	0.04524	0	0	0	0	0
GMFC-MLD 2	0	0	0.95900	0.04100	0	0	0	0
GMFC-MLD 3	0	0	0	0.95900	0.04100	0	0	0
GMFC-MLD 4	0	0	0	0	0.959003586	0.04100	0	0
GMFC-MLD 5	0	0	0	0	0	0.964563099	0.03544	0
GMFC-MLD 6	0	0	0	0	0	0		
Dead	0	0	0	0	0	0	0	1

The mean time from GMFC-MLD 6 to death was calculated using parametric extrapolation of the natural history data as per Figure 1 below.

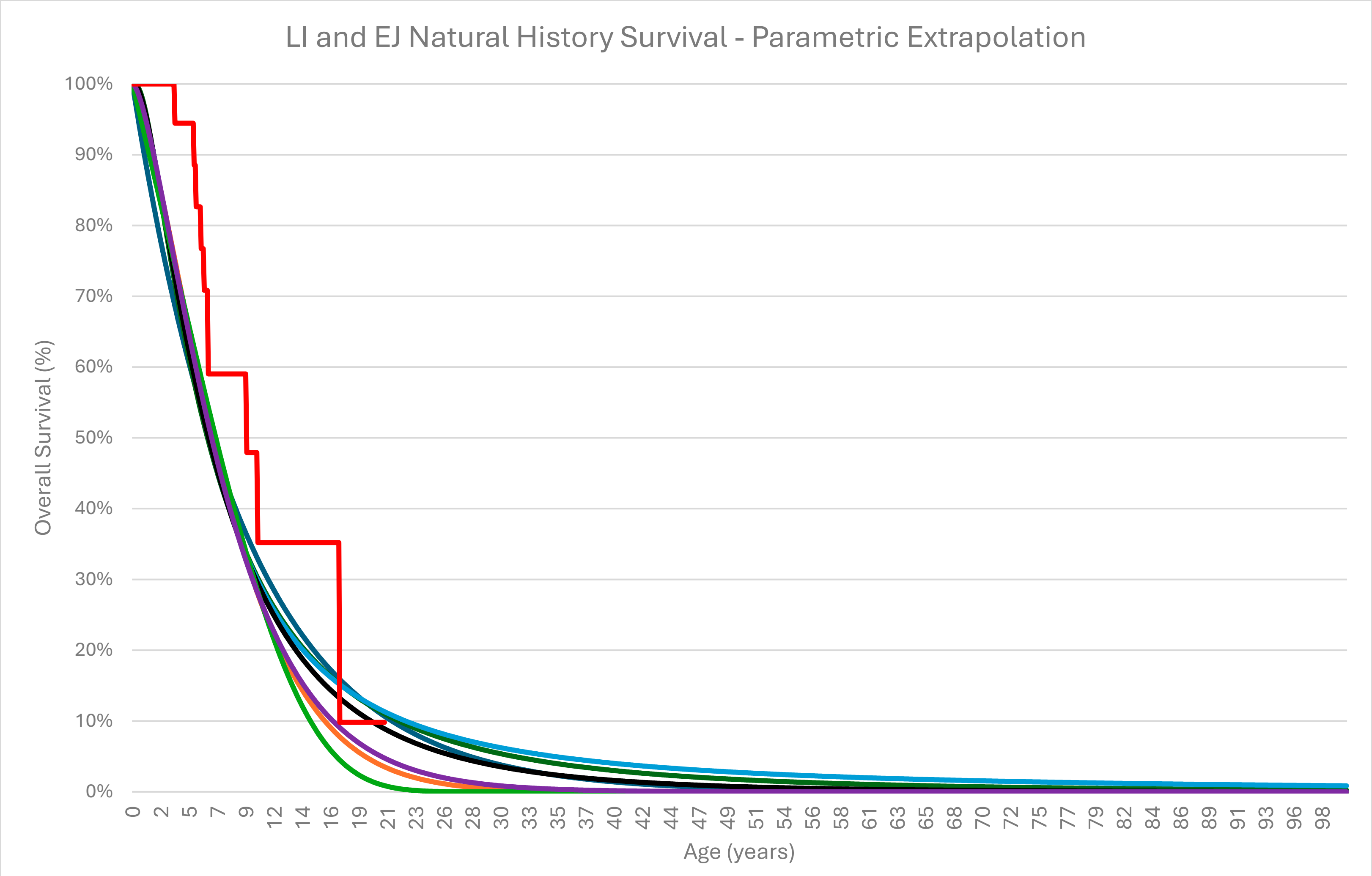

Figure S1: Mean time from GMFC-MLD 6 to death using parametric extrapolation

Full responders and stable partial responders for each MLD sub-type remain in the stabilised health state they were at last follow-up for the duration of the time horizon (lifetime) as per the proportions outlined in Table 5 below.

Table S5: Classification of response used to calculate overall time in GMFC-MLD health state at the terminal nodes of the decision tree for all MLD sub-types for the screening and no screening arms of the model base case (Figure 3 in manuscript)

	No screening (data from clinical trial 201222; compassionate use and hospital exemption)[a]			Screening		
Responder Status	Disease Variant			Disease Variant		
	PS LI (n=15)	PS EJ (n=5)[b]	ES EJ (n=5)	PS LI	PS EJ	ES EJ
Percentage of Full Responders	33%	60%	0%	100%	100%	100%
Percentage of Partial Responders stabilising at GMFC 1	33%	0%	20%	0%	0%	0%
Percentage of partial responders stabilising at GMFC 2	27%	0%	20%	0%	0%	0%
Percentage of partial responders stabilising at GMFC 3	0%	0%	20%	0%	0%	0%
Percentage of partial responders stabilising at GMFC 4	0%	0%	20%	0%	0%	0%
Percentage of unstable partial responders who have continued disease progression but at a slower rate than NHx	7%	40%	20%	0%	0%	0%

[a] At the time of the NICE HST appraisal, data from the cryopreserved formulation of arsa-cel study (N=10) was deemed by the ERG to have insufficient follow-up (< 2 years) to be included in the estimates of efficacy.

[b] One PS EJ patient died of a cerebral ischaemic infarction (unrelated to arsa-cel) 415 days after GT and before the predicted onset of symptoms. The ERG classified them as an unstable partial responder, however it is difficult to adequately classify their response status in the model and they could potentially be excluded from the analysis.
